# Identification of Reference Gene for Quantitative Gene Expression in Early-Term and Late-Term Cultured Canine Fibroblasts Derived from Ear Skin

**DOI:** 10.3390/ani14182722

**Published:** 2024-09-20

**Authors:** Sang-Yun Lee, Yeon-Woo Jeong, Yong-Ho Choe, Seong-Ju Oh, Rubel Miah, Won-Jae Lee, Sung-Lim Lee, Eun-Yeong Bok, Dae-Sung Yoo, Young-Bum Son

**Affiliations:** 1 Department of Theriogenology and Biotechnology, College of Veterinary Medicine, Gyeongsang National University, Jinju 52628, Republic of Korea; sy_lee@gnu.ac.kr (S.-Y.L.); yhchoego@gmail.com (Y.-H.C.); osj414@gnu.ac.kr (S.-J.O.); sllee@gnu.ac.kr (S.-L.L.); 2 Stem Cell Convergence Research Center, Korea Research Institute Bioscience and Biotechnology (KRIBB), Daejeon 34141, Republic of Korea; 3 Department of Companion Animal and Animal Resources Science, Joongbu University, Geumsan 32713, Republic of Korea; doctorj1@joongbu.ac.kr; 4 Department of Obstetrics, College of Veterinary Medicine, Chonnam National University, 300 Yonbongdong, Buk-gu, Gwangju 61186, Republic of Korea; rubelsau26@gmail.com; 5 Department of Obstetrics, College of Veterinary Medicine, Kyungpook National University, Daegu 41566, Republic of Korea; iamcyshd@knu.ac.kr; 6 Research Institute of Life Sciences, Gyeongsang National University, Jinju 52828, Republic of Korea; 7 Division of Animal Diseases & Health, National Institute of Animal Science, Rural Development Administration, Wanju 55365, Republic of Korea; 8 Departement of Veterinary Epidemiology, College of Veterinary Medicine, Chonnam National University, 300 Yonbongdong, Buk-gu, Gwangju 61186, Republic of Korea; shanuar@jnu.ac.kr

**Keywords:** canine, fibroblasts, long-term expansion, qRT-PCR, reference gene

## Abstract

**Simple Summary:**

This study aimed to identify stable reference genes in early-passage and late-passage cultured canine skin fibroblasts. The early-passage fibroblasts retained their spindle-shaped morphology, exhibited a short doubling time, and had low β-galactosidase activity. In contrast, the late-passage fibroblasts displayed an elongated morphology, a prolonged doubling time, and elevated β-galactosidase activity. To assess the stability of the reference genes, the Ct values obtained using qRT-PCR were analyzed using three algorithms: geNorm, NormFinder, and BestKeeper. As a result, HPRT1, YWHAZ, and GUSB were identified as the most stable reference genes across all three algorithms in canine skin fibroblasts. When comparing early-passage to late-passage fibroblasts, the normalization of Vimentin expression using both stable and unstable reference genes showed a decrease in late-passage cells. Although the use of less stable reference genes did not result in a significant difference in Vimentin expression, the use of stable reference genes revealed a significant difference. This study provides a foundation for the further application of RT-qPCR in the gene expression analysis of long-term expanded canine skin fibroblasts.

**Abstract:**

Fibroblasts are cells that reside within the fibrous or loose connective tissues of most mammalian organs. For research purposes, fibroblasts are often subjected to long-term culture under defined conditions, during which their properties can significantly change. It is essential to understand and document these changes to obtain reliable outcomes. For the quantification of specific gene expressions, the most reliable and widely used technique is quantitative real-time polymerase chain reaction (qRT-PCR). Here, we assessed the impact of a reference gene’s stability on a qRT-PCR analysis of long-term cultured canine skin fibroblasts. After successfully isolating the fibroblasts from canine skin tissues, they were cultured and evaluated for proliferation and β-galactosidase activity at different passage numbers. With extended culture, the fibroblasts showed a long doubling time and elevated β-galactosidase activity. Using three widely used algorithms, geNorm, Normfinder, and Bestkeeper, we identified HPRT1, YWHAZ, and GUSB as the most stable reference genes for both early- and late-passage fibroblasts. Conventional reference genes such as GAPDH were found to be less stable than those genes. The normalization of Vimentin by the stable genes showed statistical differences, whereas normalization by an unstable gene did not. Collectively, this study indicates that using stable reference genes is essential for accurately and reliably measuring gene expression in both early- and late-passage fibroblasts. These findings provide valuable insights into internal controls for gene expression studies and are expected to be utilized for analyzing gene expression patterns in molecular biology research.

## 1. Introduction

Fibroblasts are mesenchymal cells in soft connective tissues that play a crucial role in maintaining homeostatic functions. They are typically found in a quiescent state and play a key role in the synthesis and turnover of extracellular matrix (ECM) components [1]. They also produce connective tissues and act as progenitors for other specialized mesenchymal cell types, including adipocytes and osteoblasts. These cell types contribute to adult homeostasis, embryonic development, and the processes of tissue remodeling and injury repair [2]. Fibroblasts are widely used in molecular biology research, including cell culture models, genetic studies, and reprogramming studies [3,4,5]. They have also shown great potential for the development of genetically modified animals [6,7]. Specifically, fibroblasts are considered ideal for conserving endangered species and maintaining the germplasm of animals with superior genetics because they are used as nuclear donors in the somatic cell nuclear transfer procedure. Nevertheless, the various applications of fibroblasts are limited by their short lifespan [8]. In this regard, it is crucial to understand and characterize the changes in cellular properties experienced by fibroblasts cultured long-term in experimental studies.

Studies on gene expression have identified gene regulatory networks underlying cellular functions and enabled a quantitative analysis of gene expression, making them crucial to cellular biology research [9]. For gene expression quantification, a quantitative real-time polymerase chain reaction (qRT-PCR) is considered the most reliable and widely used technique [10] because it does not require post-PCR handling. To compare the expression of the gene of interest (GOI) across different experiments using this technique, normalization with a suitable reference gene is required. Regardless of the experimental conditions, reference genes are expected to show consistent expression in cells of interest [11,12,13]. However, it has been reported that this is not always the case, as no single reference gene can be universally applied to all experimental conditions [14,15]. Thus, it is crucial to select a suitable reference gene for studies involving various research conditions, such as cell expansion that requires long-term culture. In human cells, the changes in cell properties during long-term culture have been investigated to identify a suitable reference gene [16]. However, the impact of a reference gene’s stability in long-term cultured canine skin fibroblasts has not been investigated thus far.

The common reference genes used for gene expression analysis are glyceraldehyde-3-phosphate dehydrogenase (*GAPDH*), β−actin (*ACTB*), and TATA−binding protein (*TBP*) [17,18,19]. However, several studies have reported that these conventional reference genes may be unsuitable for RNA transcription analysis [20,21]. Therefore, we used three algorithms to identify reference genes that are more stable and suitable than the conventional ones. First, the average pair-wise variance of the expression of the candidate reference gene was compared to that of all other evaluated reference genes using geNorm. Next, an analysis of variance was used to compare the Normfinder in all sample cycle threshold values (Ct values) and assess expression stability. The geometric mean of the Ct values in each sample’s reference gene was pooled to create Bestkeeper indices. Finally, the stability of the reference gene was ranked based on pairwise comparisons of the Bestkeeper indices and Pearson correlation coefficients [22].

## 2. Materials and Methods

### 2.1. Fibroblast Isolation and Culture

Three male mixed-breed dogs, aged 1 year old and weighing 20–25 kg, were used in this study. All animal experiments were performed according to the animal study guidelines approved by the ethics committee of the Abu Dhabi Biotech Research Foundation, South Korea (Permit no. C-20-01). Before anesthesia, the dogs were fasted for 12 h. Propofol (1 mg/kg; Myungmoon Pharm., Seoul, Republic of Korea) was intravenously administered for initial anesthesia, which was maintained by 2% isoflurane inhalation. Under aseptic conditions, skin tissues on the left flank area were collected via punch biopsy (twice). These tissues were sliced into 1–2 mm^2^ pieces and explanted to a culture dish. The tissues were cultured in advanced Dulbecco’s modified Eagle’s medium (ADMEM) supplemented with 10% fetal bovine serum (FBS), 100 µg/mL streptomycin, and 100 IU/mL penicillin at 37 °C in a humidified atmosphere with 5% CO_2_. After removing the tissues, the attached fibroblasts were expanded in fresh culture medium. The culture medium was changed twice a week. Upon reaching 70–80% confluence, the cells were harvested using 0.25% trypsin-ethylene-diamine-tetra-acetic acid (Trypsin-EDTA) solution with centrifugation at 300× *g* for 5 min. The harvested cells were cultured further until they reached passage number 8.

### 2.2. Analysis of Proliferation Potential in Long-Term Expanded Fibroblasts

To evaluate the proliferation of fibroblasts, the population of doubling time (PDT) was assessed following a previously reported protocol [23]. At each passage, the fibroblasts were seeded into a 6-well culture plate. Every 48 h, the cells were detached and counted using a hematocytometer. PDT was calculated using the following equation:

PDT = log2 + T/(logNH − log NI)

where T is the culture time, logNH is the harvest cell number, and log NI is the initial cell number.

### 2.3. Senescence-Associated β-Galactosidase Assay in Long-Term Expanded Fibroblasts

A Mammalian β-Galactosidase Assay Kit (ThermoFisher, Rockford, IL, USA) was used to assess the β-galactosidase activity (SA-*β*-gal). At each passage, 5 × 10^5^ fibroblasts were reacted with M-PER reagent for 10 min. The cells were then centrifuged for 10 min at 27,000× *g*. Next, 50 μL of the supernatant was transferred to a 96-well plate and incubated with the β-galactosidase reagent for 30 min at 37 °C. The optical density at 405 nm was measured with a microplate reader (VersaMax™, Molecular Devices, CA, USA). 

### 2.4. Candidate Reference Genes and Primers

The candidate reference genes were selected based on their various intracellular functional activities and in accordance with previous studies [11,12,13,24]. The reference gene primers were designed using Primer 3 Plus software. Using OligoAnalayzer 3.1 software, we confirmed that the primers did not form hairpins nor homodimers. Additionally, we verified that no interactions, such as heterodimer formation, occurred between the forward and reverse primers. To analyze PCR efficiency, a standard curve was constructed for each primer based on their Ct values from a four-fold serial dilution of cDNA. Standard curve parameters included standard curve slope (M), intercept (B), and PCR efficiencies (E). Correlation coefficients (R^2^) were calculated using RoterGene Q Series Software 2.1.0 (Qiagen, Hilden, Germany). The primer information (gene symbol, nucleotide sequences, amplicon sizes, accession number, and PCR efficiencies) is described in Table 1. 

### 2.5. RNA Isolation, cDNA Synthesis, and qRT-PCR

At each passage, RNA was extracted using RNeasy Mini Kit (Qiagen). To prevent DNA contamination, RNase-free DNase treatment was performed prior to isolation. The concentration and purity of RNA were assessed using an OPTIZEN NanoQ Lite spectrophotometer. For cDNA synthesis, 1 μg of total RNA was reacted with Omniscript Reverse Transcription Kit (Qiagen) for 1 h at 37 °C. For PCR amplification, qRT-PCR was performed with a reaction mixture containing 2X SYBR Green mix (Qiagen), 2 μL cDNA, 0.7 μM forward primer, and 0.7 μM reverse primer. The reaction was run using Rotor Gene Q qRT-PCR (Qiagen) as follows: pre-denaturation, denaturation, and a combined annealing/extension step with minor modifications to the manufacturer’s protocol. Pre-denaturation was conducted at 95 °C for 2 min; 45 PCR cycles were conducted at 95 °C for 10 s and at 60 °C for 6 s. The melting curve showed temperature changes from 60 °C to 95 °C by 1 °C/s and cooling at 40 °C for 30 s. Rotor-Gene Q Series software (Qiagen) was used to obtain the Ct values and melting curves. The expected product size and absence of non-specific amplification of all qRT-PCR products were confirmed by gel electrophoresis. 

### 2.6. Analysis of Stable Reference Gene Expression

To analyze stable reference genes, the following algorithms were used: geNorm, Normfinder, and Bestkeeper. The geNorm algorithm estimated the stability of gene expression (M value). The gene with the largest M value, indicating the lowest stability, was excluded from the pool. The pool was continuously assessed and redefined based on M values until the last two candidates were left as the most stable reference genes. Furthermore, geNorm determined the ideal number of reference genes for the normalization factor (NF) by continuously calculating the pairwise variance (V_n/n+1_) between successively ranked NF (NF_n_ and NF_n+1_) values [17]. To minimize excessive number of reference genes during normalization, Pearson’s correlations of NFs were assessed using SPSS to determine the association between the four most stable reference genes (NF_4_) and the ideal number of reference genes (NF_opt_). Normfinder, which is based on the Visual Basic for Applications (VBA) platform, was used to calculate both intra- and inter-group variations to identify the most stable reference gene represented by the lowest value. Additionally, Normfinder suggested the optimal pair of genes for normalization [25]. Bestkeeper was used for comparative analysis based on Pearson’s pairwise correlations among all reference genes, and the standard deviations (SDs) were calculated. The reference genes with SD values greater than 1.0 were considered unsuitable, while those with low SD values were considered stable and suitable [26]. 

### 2.7. The Use of Different Reference Genes in the Normalization of GOI

To analyze the impact of the reference gene’s stability, the GOI was normalized based on the most stable reference gene identified, alongside conventional reference genes with lower stability. *Vimentin* expression was evaluated using qRT-PCR as described above. The relative mRNA level of *Vimentin* was normalized to the aforementioned reference genes using RotorGene Q Series software. 

### 2.8. Statistical Analysis

SPSS version 23 (SPSS Inc., Chicago, IL, USA) software was used for data analysis. The statistical differences among all groups were determined by one-way analysis of variance (ANOVA) with Tukey’s post hoc test. All data are presented as mean ± SD; *p* < 0.05 was considered statistically significant. 

## 3. Results

### 3.1. Cell Properties of Long-Term Expanded Fibroblasts

**Figure 1:**
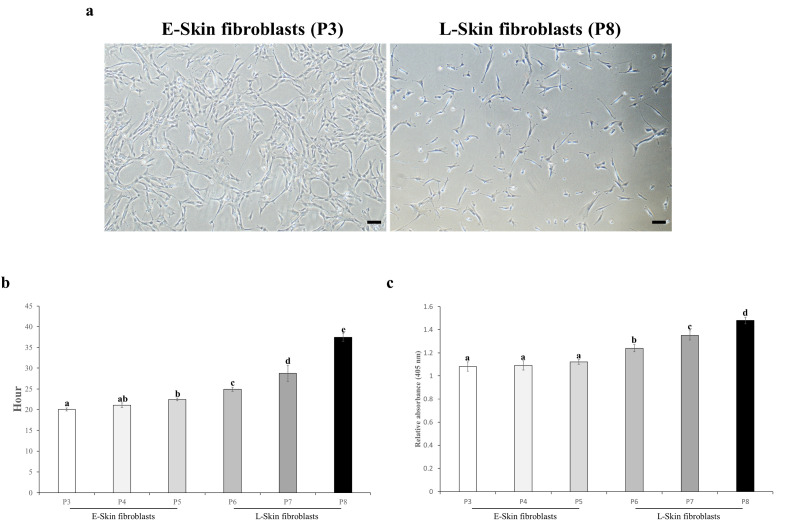
Comparative canine skin fibroblasts in the early passage and alternation of their properties in the late passage. (**a**) A spindle-shaped morphology in the long-term expanded canine skin fibroblasts at early (E-skin fibroblasts) and late (L-skin fibroblasts) passages. The L-skin fibroblasts appeared as more elongated cells compared to the E-skin fibroblasts. Scale bar = 50 μm. (**b**) The in vitro proliferation capacity of the skin fibroblasts using the population of doubling time (PDT). The PDT was measured by counting the cell number every 48 hr in each passage. (**c**) Senescence-associated *β*-galactosidase (SA-*β*-gal) activity in the E-skin fibroblasts and L-skin fibroblasts. Absorbance was measured at 405 nm. The data are presented as the mean ± SD. Lettered subscripts indicate statistical differences between groups (*p* < 0.05).

Fibroblasts were successfully isolated from the resected canine skin tissues. At early and late passages, the fibroblasts displayed a spindle-shaped morphology [27] in which the late-passage fibroblasts appeared as elongated cells (Figure 1a). These cells were analyzed for proliferation and SA-*β*-gal activity at passage numbers 3, 4, 5, 6, 7, and 8. During passages 3, 4, and 5 (early or “E-skin fibroblasts”), the cells displayed consistently short doubling times. By passages 6, 7, and 8 (late or “L-skin fibroblasts”), the cells displayed significantly longer doubling times compared to the E-skin fibroblasts (Figure 1b). In terms of cellular senescence represented by SA-*β*-gal activity, the L-skin fibroblasts showed significantly elevated activity compared to the E-skin fibroblasts (Figure 1c). RNA was successfully isolated at passage numbers 3, 4, 5, 6, 7, and 8. The quality of the isolated RNA was confirmed to be appropriate by analyzing the absorbance ratios at 230, 260, and 280 nm (Supplementary Table S1).

### 3.2. Evaluation of Primer Efficiency, Amplicon Size, and Ct Values

**Figure 2:**
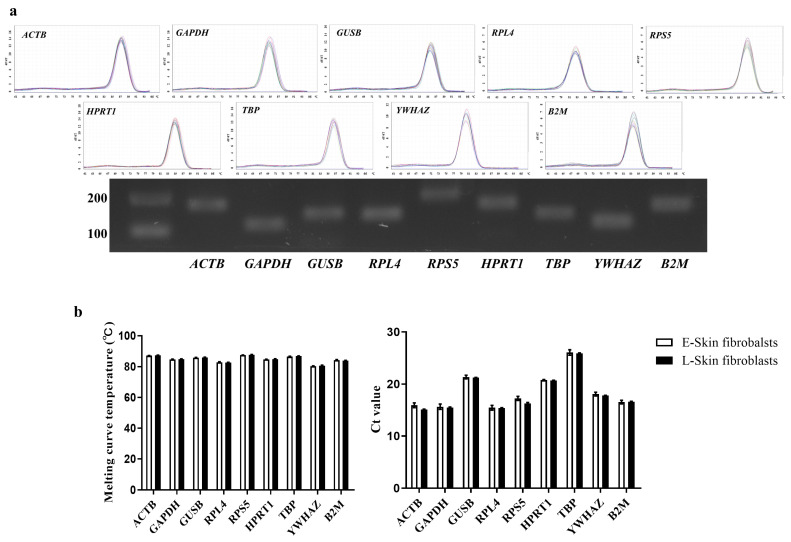
An examination of the melting curve, amplicon size, and Ct value of the candidate reference genes. (**a**) A melting curve analysis and agarose gel electrophoresis were used to verify the specificity of primers and the amplicon lengths of the 9 selected reference genes. Electrophoresis using 1% agarose gel showed the expected product size without non-specific amplification. (**b**) The Ct values of the 9 selected reference genes in canine skin fibroblasts at early passage (E-skin fibroblasts) and late passage (L-skin fibroblasts). The data are presented as the mean ± SD.

Primer efficiency was determined from a four-fold serial dilution of cDNA, resulting in E values ranging from 0.97 to 1.02 and R^2^ values ranging from 0.991 to 0.999; these values confirm the suitability of the candidate reference genes for qRT-PCR. A melting curve analysis was conducted to validate the primer specificity in which each primer amplification yielded a high single-peak melting curve. In addition, gel electrophoresis of the amplicons confirmed their expected sizes and the absence of non-specific products such as hairpin and dimers (Figure 2a). To analyze the difference in transcription level between the E- and L-skin fibroblasts, all reference genes’ Ct values were analyzed using qRT-PCR (Figure 2b). Although varying Ct values were yielded, no significant difference was observed for the long-term expanded fibroblasts.

### 3.3. An Analysis of the Most Stable Reference Gene Using geNorm

**Figure 3:**
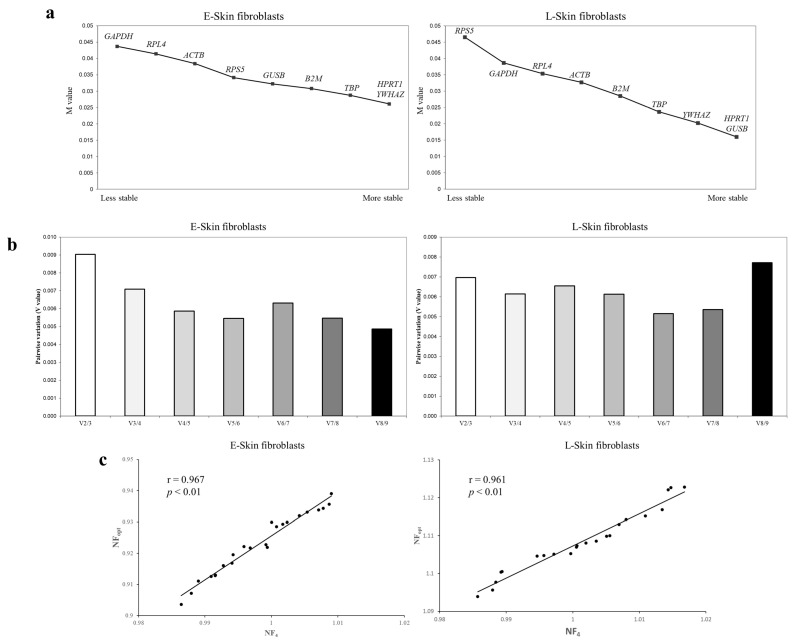
An analysis of the most stable reference gene used for geNorm. (**a**) The stability ranking of these reference genes for normalization was determined based on their M values. The most stable reference gene is indicated on the right side of the graph. (**b**) The optimal number of reference genes for normalization (NF_opt_). The NF_opt_ value was suggested to be 8 and 6 reference genes by pairwise variation (V_8/9_ and V_6/7_) in the canine skin fibroblasts at early (E-skin fibroblasts) and late passage (L-skin fibroblasts), respectively. (**c**) A correlation was found between the NF_opt_ and NF4 values. Pearson’s correlation was used to analyze the correlation between NF_opt_ and NF_4_. NF_4_ means the four most stable reference genes for normalization.

The Ct values of the E- and L-skin fibroblasts were analyzed using geNorm. The M value was used to rank the stability of candidate reference genes, and the less stable candidates were progressively removed from the pool. All candidates showed M values of less than 1.5, indicating that they were all suitable reference genes. The analysis indicated that *HPRT1*, *YWHAZ*, and *TBP* were the most stable reference genes in the E-skin fibroblasts. For the L-skin fibroblasts, *HPRT1*, *GUSB*, and *YWHAZ* were the most stable candidates. In particular, conventional reference genes, such as *GAPDH* and *ACTB*, were found to be unstable in both the E- and L-skin fibroblasts (Figure 3a). Furthermore, pairwise variations indicated that the NF_opt_ values of the E- and L-skin fibroblasts were V_8/9_ and V_6/7_, respectively (Figure 3b). The optimal number of reference genes was proposed to be eight and six for normalization in both the E- and L-skin fibroblasts, respectively. To avoid the excessive use of 6–8 reference genes, the correlation of NF using an optimal number was analyzed. A high correlation (r > 0.96) between the NF_4_ and NF_opt_ values was observed for both the E- and L-skin fibroblasts (Figure 3c).

### 3.4. An Analysis of the Most Stable Reference Gene Using Normfinder

**Figure 4:**
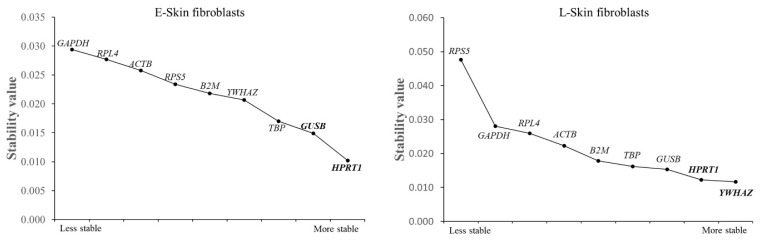
An evaluation of the reference genes’ stability using Normfinder. The most stable reference gene is ranked on the right side of the graph, while the most unstable reference gene is ranked on the left side. The best combination of two reference genes is shown in bold letters.

According to Normfinder, HPRT1 and YWHAZ were identified as the most stable reference genes in the E- and L-skin fibroblasts, respectively. In addition, it was shown that GUSB with HRPT1 and HPRT1 with YWHAZ were the best combinations of reference genes in the E- and L-skin fibroblasts, respectively. Similar to the geNorm analysis, HPRT1 and TBP were identified as the most stable reference genes in the E-skin fibroblasts. Furthermore, HPRT1, GUSB, and YWHAZ were consistently identified as the most stable reference genes in the L-skin fibroblasts. On the other hand, the conventional reference genes (GAPDH and ACTB) were again found to be unstable according to Normfinder (Figure 4).

### 3.5. An Analysis of the Most Stable Reference Gene Using Bestkeeper

**Figure 5:**
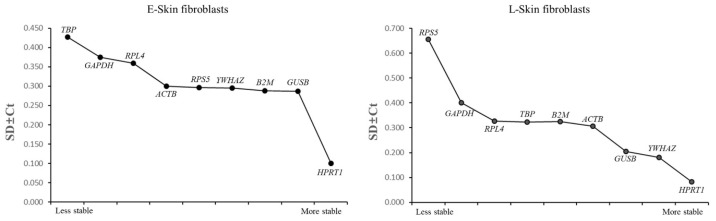
An assessment of the most stable reference gene using Bestkeeper. The ranking of the stability (SD ± Ct) of the reference gene was assessed from the least stable reference gene, shown on the left side of the graph, to the most stable reference gene, shown on the right side.

To investigate the expression stability of the reference genes, Bestkeeper was used to estimate their SD values. No reference gene showed SD > 1.0, indicating their reliability for this study. *GUSB* and *HPRT1* were the most stable reference genes, showing the lowest SD ± Ct values in the E-skin fibroblasts. Similarly, *HPRT1* and *YWHAZ* displayed the lowest SD ± Ct values in the L-skin fibroblasts (Figure 5). The slight variations in the stability rankings of the reference genes across geNorm, Normfinder, and Bestkeeper may be attributed to their different calculation algorithms.

### 3.6. GOI Normalization Using the Most Stable Reference Gene Identified

**Figure 6:**
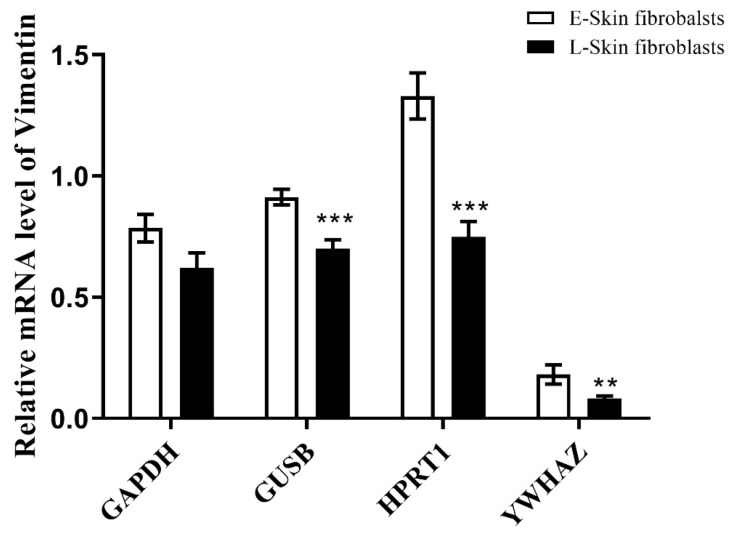
The normalization of the estimated reference gene. The normalization of the relative mRNA level using stable reference genes (*GUSB*, *HPRT1*, and *YWHAZ*) or a less stable reference gene (*GAPDH*). The expression of *Vimentin* was normalized by GUSB, HPRT1, YWHAZ, and GAPDH to demonstrate the effect of the reference gene’s stability. The data are presented as the mean ± SD. Significant (** *p* < 0.01 and *** *p* < 0.001) differences in the *Vimentin* expression level between canine skin fibroblasts at early (E-skin fibroblasts) and late passage (L-skin fibroblasts) are shown with an asterisk.

The three algorithms indicated that *HPRT1*, *YWHAZ*, and *GUSB* are the most stable reference genes in the pool. These genes and a conventional reference gene (*GAPDH*) with lower stability were used for the normalization of *Vimentin*, a common expression marker of fibroblasts, to confirm their impact. When normalized using both stable and unstable reference genes, the L-skin fibroblasts exhibited decreased expression compared to the E-skin fibroblasts. While *GAPDH* did not show a significant difference in *Vimentin* expression, *HPRT1*, *YWHAZ*, and *GUSB* yielded a significant difference (Figure 6).

## 4. Discussion

In molecular biology, RT-PCR is considered the most reliable and widely utilized method for quantifying the mRNA levels of specific genes [10]. Using stable reference genes is crucial in qRT-PCR to accurately detect changes in gene expression. Previous studies involving bovine and human fibroblasts have been conducted to identify the most stable reference genes for such analysis [28,29]. Inspired by these studies, we aimed to identify the most stable reference genes for analyzing gene expression in long-term expanded skin fibroblasts. For this, we combinatorically used three widely used algorithms (geNorm, Normfinder, and Bestkeeper) to identify the best candidate for qRT-PCR normalization.

The long-term expanded fibroblasts exhibited limited proliferation due to replicative senescence, similar to that observed in other somatic cell types [30,31]. In somatic cells, senescence impacts proliferative capacity, cell size, SA-*β*-gal activity, and metabolism and alters the gene expression profile, including the gene regulatory network [32,33]. In this study, skin fibroblasts maintained a mesenchymal spindle-shaped morphology at both early and late passages. However, upon long-term culture, the L-skin fibroblasts exhibited an increased cell size and irregular morphologies. Additionally, these cells showed significant increases in the PDT and SA-*β*-gal activity. Overall, we observed that the long-term culture of fibroblasts results in altered cellular properties. These changes are consistent with previous observations of fibroblasts and other mesenchymal lineage cell types [34,35,36].

Reference genes, such as *GAPDH* and *ACTB*, function as basic cell survival factors and play a crucial role in maintaining cellular functions [16]. *GUSB* is a key lysosomal enzyme that plays a crucial role in the degradation of glucuronate-containing glycosaminoglycan [37]. *RPL4* and *RPS5* are structural components of the ribosome [38]. *HPRT1* catalyzes the conversion of hypoxanthine and guanine to their respective mononucleotides, playing a crucial role in purine salvage pathways [26]. *YWHAZ*, a member of the 14-3-3 protein family, functions as a central hub protein in various signal transduction pathways and plays a critical role in tumor progression [24]. Based on this, the candidate reference genes were selected from those traditionally and widely used in qRT-PCR experiments. For qRT-PCR, it has been demonstrated that the stability of reference genes can vary under different experimental conditions, with no single reference gene being universally applicable to all conditions. Factors like the primer size and amplicon length have been shown to play important roles in reference gene stability [36]. To minimize their potential impacts, we utilized primers with sizes between 22 and 24 and amplicons with sizes between 100 and 200 base pairs. As shown in Figure 2b, the reference gene expression levels can be impacted by experimental conditions. Previous studies have demonstrated that culture duration can influence the expression levels and trends in reference genes, which are generally regarded as fundamental factors for cell survival [16,39]. Therefore, choosing non-validated or unstable reference genes for the normalization of the GOI may result in inconsistent or misleading outcomes [40,41]. Hence, selecting the most stable reference genes under specific experimental conditions is considered crucial for qRT-PCR analysis.

To analyze the stability of reference genes, the Ct values obtained using qRT-PCR were assessed using geNorm, NormFinder, and BestKeeper. Consequently, *HPRT1*, *YWHAZ*, and *GUSB* were determined to be the most stable reference genes in canine skin fibroblasts. In contrast, conventional reference genes such as *GAPDH* and *ACTB* were observed to be less stable than those genes. In previous studies, *HPRT1* was identified as a stable reference gene in canine samples across various experimental conditions, including healthy and dystrophic muscles, as well as irradiated skin tissues [22,42]. In healthy and dystrophic canine brains, *YWHAZ* with *SDHA* and *UBC* were identified to be the most suitable reference genes for normalizing gene expression [43]. Additionally, *GUSB* and *PSMB6* were found to be the most stably expressed reference genes in canine soft tissue sarcomas, as determined using geNorm [44]. In terms of conventional reference genes, several studies on the canine hindbrain and canine oral tumors determined that *GAPDH* and *ACTB* are stable for their analyses, respectively [45,46]. In contrast, studies on other canine tissues determined that these gene are unstable for their analyses [36,47]. Collectively, this indicates that different experimental conditions, such as cell source, donor variability, treatment, differentiation induction, and cell expansion, can profoundly affect the stability of reference genes.

To demonstrate the potential controversy when using unstable reference genes, we normalized the GOI based on both stable and unstable reference genes. In the E- and L-skin fibroblasts, the normalization of *Vimentin* was conducted using the most stable reference genes identified (*HPRT1*, *YWHAZ*, and *GUSB*) along with a conventional reference gene (*GAPDH*) with lower stability according to the three algorithms. *Vimentin* is a widely recognized marker of fibroblasts [48,49]. *Vimentin* is involved in a variety of cellular processes, including cell adhesion, migration, invasion, signaling, differentiation, cytoskeletal reorganization, and the modulation of cell shape and plasticity [40,41,42,43,44,45,46,47,48,49,50,51,52,53]. Previous studies have shown that *Vimentin* expression changes with senescence [54]. In this study, the normalization of *Vimentin* based on both stable and unstable reference genes led to decreased expression in the L-skin fibroblasts compared to the E-skin fibroblasts. Despite the similar trends shown by stable and unstable reference genes for normalization, a statistical analysis revealed significant differences between them. The normalization process using stable reference genes revealed significant differences in gene expression, whereas using the unstable reference gene did not show such differences. This indicates the importance of validating reference genes before normalization for qRT-PCR analysis [16].

## 5. Conclusions

In conclusion, *HPRT1*, *YWHAZ*, and *GUSB* were identified as the most stable reference genes from the pool of nine genes. Conventional reference genes such as *GAPDH* and *ACTB* were identified to be less stable under the same experimental conditions. Also, it was demonstrated that *HPRT1* is the most stable reference gene for analyzing both early- and late-passage canine skin fibroblasts. This study will serve as a foundation for RT-qPCR-based research on long-term expanded canine skin fibroblasts.

## Figures and Tables

**Table 1:** Information on candidate reference genes and *Vimentin*.

	Information of Primers		Standard Curve Parameters				
Gene Name (Symbol)	Sequence	Base Pair	Accession	R^2^	M	B	E
Beta-actin (*ACTB*)	F: GCACTCTTCCAACCTTCTTTCC R: GCTGTGATTTCCTTCTGCATCC	179	AF021873.2	0.993	−3.489	34.252	1.01
Glyceraldehyde-3-phosphate dehydrogenase (*GAPDH*)	F: GGAGAAAGCTGCCAAATATGACG R: ACTGTTGAAGTCACAGGAGACC	118	NM_001003142.2	0.991	−3.436	35.514	0.98
Beta-glucuronidase (*GUSB*)	F: ATCTGTAGTCATGTGGTCTGTAGC R: GGTCTGCTTCATAGTTGGAATTGG	149	AF019759.1	0.996	−3.332	33.257	0.99
Ribosomal protein 4 (*RPL4*)	F: AATGAGAAACCGTCGTCGTATCC R: GGAGCAAGTTTCAGAATGTTCAGC	141	NM_001252409.1	0.992	−3.355	39.041	1.01
Ribosomal protein S5 (*RPS5*)	F: TGAAGGAGAAGTATGCCAAGTACC R: GAGCAGATGGATGATCTCGAAGG	188	XM_533568.5	0.995	−3.435	39.145	0.97
Hypoxanthine phosphoribosyl transferase 1 (*HPRT1*)	F: GACTGAAGAGCTACTGTAATGACC R: TCTTTGGATTATGCTCCTTGACC	168	NM_001003357.2	0.996	−3.412	36.915	0.98
TATA box-binding protein (*TBP*)	F: ATCTGGTATCCCTTACGCTTCG R: GCAAGAGAGTCTGGTTTGTTTCC	137	XM_849432.4	0.995	−3.498	36.972	1.02
Tyrosine 3-monooxygenase/tryptophan 5-monooxygenase activation protein, zeta polypeptide (*YWHAZ*)	F: GTGAAGAGTCATACAAAGACAGCA R: CCCTCCTTCTCCTGCTTCAG	110	XM_014118550.1	0.992	−3.511	37.145	1.01
Beta-2 microglobulin (*B2M*)	F: AGATGAAAGCAGAACAGACAGACC R: GTTGTCTCGGTCCCACTTAACG	161	JQ733515.1	0.999	−3.475	35.221	0.99
*Vimentin*	F: AAGTTTGCCGACCTCTCTGA R: TTCGACGGCAAAGTTCTCTT	183	NM_001287023.1				

## Data Availability

The data presented in this study are available within the article.

## Supplementary Materials

The following supporting information can be downloaded at: https://www.mdpi.com/article/10.3390/ani14182722/s1, Table S1: Quality and quantity of RNA.
