# Supplementary material for: Identification of Reference Gene for Quantitative Gene Expression in Early-Term and Late-Term Cultured Canine Fibroblasts Derived from Ear Skin
Source: Animals (Basel). 2024 Sep 20;14(18):2722. doi: 10.3390/ani14182722 (PMC11429031; doi:10.3390/ani14182722)
Supplement: Supplementary file 1 [file animals-14-02722-s001.zip › animals-3177610-supplementary.pdf]

Supplementary Table S1. Quality and quantity of RNA.

Passage	RNA quantity (ng/μl)	260/280 ratio	260/230 ratio
Passage 3	$633.21^{a} \pm 4.45$	1.98	1.99
Passage 4	$628.30^{a} \pm 4.20$	1.97	1.98
Passage 5	$626.84^{a} \pm 4.22$	2.01	2.05
Passage 6	$522.07^{b} \pm 5.29$	1.95	2.02
Passage 7	$515.72^{b} \pm 8.28$	2.03	1.91
Passage 8	$511.88^{b} \pm 6.69$	2.01	1.95

Data are represented by the mean ± SD of four independent experiments. Lettered subscripts indicate statistical differences between groups ($p < 0.05$)
